# Using a multi-state Learning Community as an implementation strategy for immediate postpartum long-acting reversible contraception

**DOI:** 10.1186/s13012-017-0674-9

**Published:** 2017-11-21

**Authors:** Carla L. DeSisto, Cameron Estrich, Charlan D. Kroelinger, David A. Goodman, Ellen Pliska, Christine N. Mackie, Lisa F. Waddell, Kristin M. Rankin

**Affiliations:** 1 Division of Epidemiology and Biostatistics, School of Public Health, University of Illinois at Chicago, 1603 Taylor St. (m/c 923), Chicago, IL 60612 USA; 2 Division of Community Health Sciences, School of Public Health, University of Illinois at Chicago, 1603 Taylor St. (m/c 923), Chicago, IL 60612 USA; 3 Maternal and Child Health Epidemiology Program, Division of Reproductive Health, National Center for Chronic Disease Prevention and Health Promotion, Centers for Disease Control and Prevention, 4770 Buford Hwy NE, MS F74, Chamblee, GA 30341 USA; 4 The Association of State and Territorial Health Officials, 2231 Crystal Drive, Suite 450, Arlington, VA 22202 USA

**Keywords:** Implementation strategies, Implementation science, Long-acting reversible contraception, Postpartum contraception, Learning collaborative

## Abstract

**Background:**

Implementation strategies are imperative for the successful adoption and sustainability of complex evidence-based public health practices. Creating a learning collaborative is one strategy that was part of a recently published compilation of implementation strategy terms and definitions. In partnership with the Centers for Disease Control and Prevention and other partner agencies, the Association of State and Territorial Health Officials recently convened a multi-state Learning Community to support cross-state collaboration and provide technical assistance for improving state capacity to increase access to long-acting reversible contraception (LARC) in the immediate postpartum period, an evidence-based practice with the potential for reducing unintended pregnancy and improving maternal and child health outcomes. During 2015–2016, the Learning Community included multi-disciplinary, multi-agency teams of state health officials, payers, clinicians, and health department staff from 13 states. This qualitative study was conducted to better understand the successes, challenges, and strategies that the 13 US states in the Learning Community used for increasing access to immediate postpartum LARC.

**Methods:**

We conducted telephone interviews with each team in the Learning Community. Interviews were semi-structured and organized by the eight domains of the Learning Community. We coded transcribed interviews for facilitators, barriers, and implementation strategies, using a recent compilation of expert-defined implementation strategies as a foundation for coding the latter.

**Results:**

Data analysis showed three ways that the activities of the Learning Community helped in policy implementation work: structure and accountability, validity, and preparing for potential challenges and opportunities. Further, the qualitative data demonstrated that the Learning Community integrated six other implementation strategies from the literature: organize clinician implementation team meetings, conduct educational meetings, facilitation, promote network weaving, provide ongoing consultation, and distribute educational materials.

**Conclusions:**

Convening a multi-state learning collaborative is a promising approach for facilitating the implementation of new reimbursement policies for evidence-based practices complicated by systems challenges. By integrating several implementation strategies, the Learning Community serves as a meta-strategy for supporting implementation.

## Background

Long-acting reversible contraception (LARC), which includes subdermal implants and intrauterine devices (IUDs), is an evidence-based method for preventing unintended pregnancies. These methods require no additional user effort once inserted and have failure rates of < 1% [1]. The most common time for receiving LARC after childbirth is at the 4- to 6-week postpartum visit; however, many women miss the postpartum visit or may become pregnant before the visit, putting them at risk for rapid repeat pregnancy and its associated adverse outcomes [2, 3]. The current versions of the US and World Health Organization Medical Eligibility Criteria for Contraceptive Use support the placement of immediate postpartum LARC, and the professional organizations of providers from the USA, UK, and Canada have recognized postpartum placement of LARC as safe, effective, and acceptable [4–8].

Although providing LARC immediately postpartum has potential for increasing access to effective contraception, barriers limit widespread adoption by birthing facilities, which include hospitals and birthing centers. In the USA, one substantial barrier is the high costs of LARC devices and insertion procedures; most payers only provide a bundled reimbursement based on the Diagnosis-Related Group (DRG) code for labor and delivery, which is not adequate to cover LARC insertion immediately after birth [9]. To address this barrier, since 2012, several state Medicaid agencies have changed their policies to allow reimbursement to birthing facilities for immediate postpartum LARC devices, and in some states, the insertion procedures, above and beyond the DRG rate for labor and delivery [10, 11]. However, experiences of states that were early adopters of a reimbursement policy change demonstrated that a policy change alone was insufficient to increase women’s access to immediate postpartum LARC and that implementation strategies were needed to bridge the gap between policy and access to this evidence-based practice [12].

Employing implementation strategies is often necessary for successfully integrating complex, evidence-based public health programs and policies into standard practice [13]. The Expert Recommendations for Implementing Change (ERIC) project recently published compilation of terms and definitions for 73 implementation strategies in order to help implementation science researchers use consistent language in their work [14]. One of the strategies the ERIC project included was the creation of a learning collaborative [14]. Previously, the Institute for Healthcare Improvement in the USA published a white paper on achieving “breakthrough” improvements through the use of a learning collaborative model, and learning collaboratives have been used to support a wide variety of public health projects, including hypertension control and breast cancer disparities [15–17].

In 2014, in partnership with the US Centers for Disease Control and Prevention (CDC), other federal agencies, and maternal and child health organizations, the Association of State and Territorial Health Officials (ASTHO) convened a multi-state learning collaborative for increasing access to immediate postpartum LARC. A cohort of six states with Medicaid policies to reimburse for immediate postpartum LARC were invited to participate in this collaborative to identify, document, and address technical assistance needs, promising practices, and barriers to immediate postpartum LARC use [18]. This collaborative, the Immediate Postpartum LARC Learning Community, hereafter referred to as simply the Learning Community, was formed based on the idea that a national-level collaborative could facilitate group learning and information sharing for a systems change approach to better apply policies supporting the use of immediate postpartum LARC [19]. In 2015, ASTHO released a call for letters of interest for any state with Medicaid payment policies in place for immediate postpartum LARC to join a second cohort of the Learning Community. Seven applicant states were accepted at that time to participate alongside the six original states. Activities of the Learning Community include a yearly in-person meeting and quarterly virtual learning sessions. The latter are organized around eight domains, which were selected after baseline interviews with the first cohort of Learning Community states to address the barriers that states face in increasing access to immediate postpartum LARC. These domains include provider training, reimbursement and sustainability, informed consent and ethical considerations, stocking and supply, outreach, stakeholder partnerships, service locations, and data, monitoring, and evaluation (Table 1) [19–21].

**Table 1:** Domains of the Immediate Postpartum LARC Learning Community [21]

Provider training	Outreach
Reimbursement and sustainability	Stakeholder partnerships
Informed consent and ethical considerations	Service locations
Stocking and supply	Data, monitoring, and evaluation

The purpose of this qualitative study was to better understand successes, challenges, and strategies used for increasing access to immediate postpartum LARC among the 13 states in the Learning Community.

## Methods

**Fig. 1:**
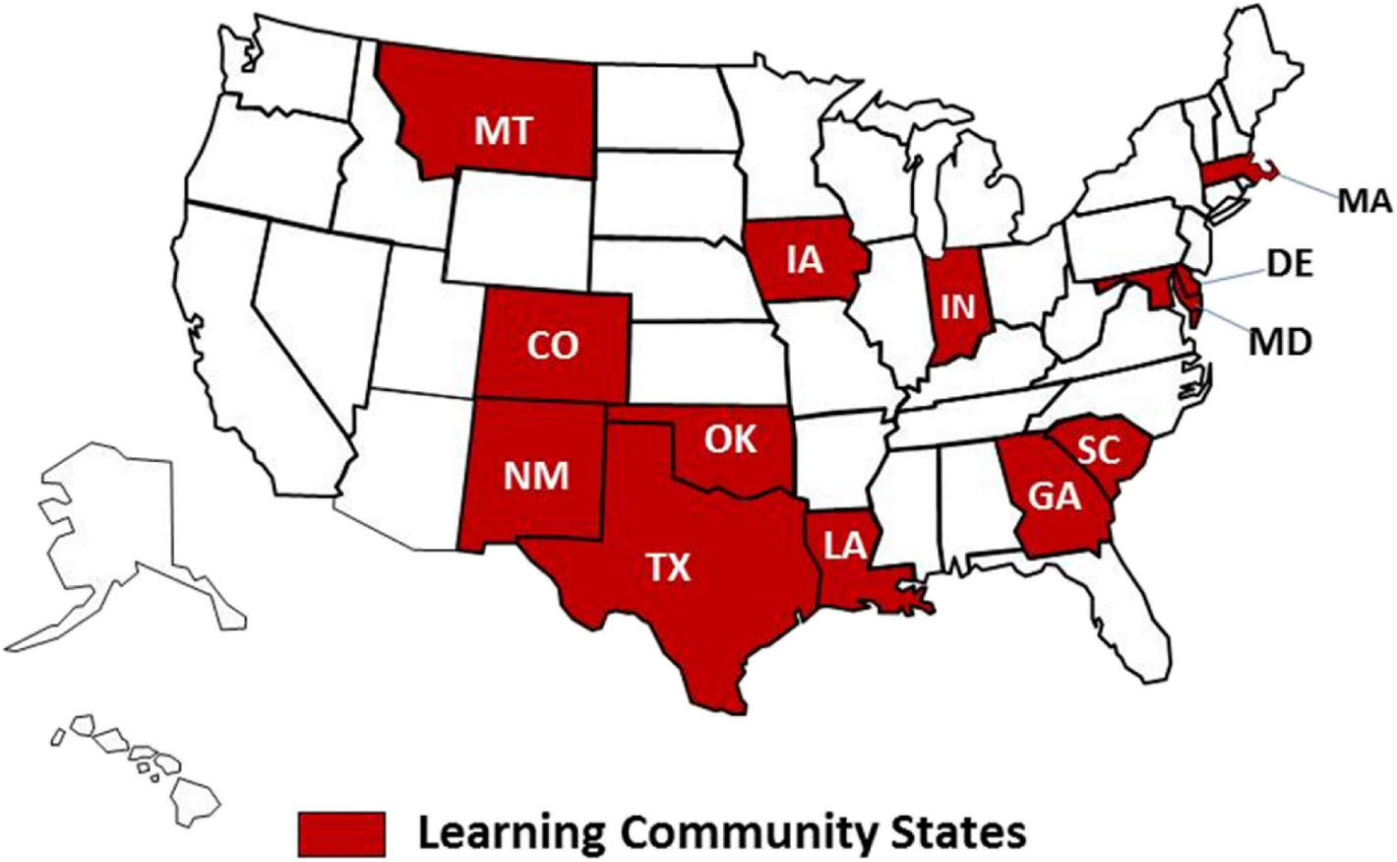
Map of the states participating in the ASTHO Immediate Postpartum LARC Learning Community

Between November 2015 and March 2016, we conducted semi-structured interviews via telephone with each of the 13 state teams participating in the Learning Community (Fig. 1). State teams were multi-disciplinary and multi-agency, consisting of three to seven people. They included Medicaid representatives, clinical providers, state health department staff, and other stakeholders. On average, four state team members joined the calls. However, in the few instances where we were unable to get multiple state team members on the phone together, we held interviews with individuals to supplement team calls. We conducted a total of 16 interviews, which included 41 participants. Each interview lasted approximately 1 h and was led by KR, the principal investigator.

We developed a semi-structured interview guide that was organized around the eight domains of the Learning Community, which are listed in Table 1 [21]. Each domain covers one aspect of increasing access to immediate postpartum LARC. Within each domain, we asked the teams to describe what their state team has done, what has helped them make their work happen, and what barriers they have faced. Each interview was audio-recorded and transcribed by a third-party vendor. For this analysis, the data were coded for facilitators, barriers, and implementation strategies. We used the compilation of implementation strategies from the ERIC project [14] as a foundation for coding the implementation strategies. Two coders (CD and CE) independently coded all 16 interviews in Dedoose version 7.0.23 (Los Angeles, CA). Following this, the two coders met to resolve coding discrepancies. Coded text was carefully reviewed by members of the primary research team (KR, CD, and CE), who then met to discuss themes in the coded data.

This project received an exemption from the Institutional Review Board at the University of Illinois at Chicago.

## Results

### Benefits of the Learning Community

One of the key themes from our qualitative data analysis was the mechanism by which the Learning Community serves as a strategy for implementing immediate postpartum LARC policies across and within states. State teams highlighted three ways in which being part of this national learning collaborative has helped them in their policy implementation work:

1. *Structure and accountability*: Being part of a national initiative with regularly scheduled meetings helps state teams get support from internal organizational leadership and prioritize their work around immediate postpartum LARC. This was explained concisely by one state team member, who said, “Knowing there’s a meeting coming up helps us stay focused on the work that we need to get done.”
2. *Validity*: For many state participants, the activities of the Learning Community require additional time and dedicated effort on top of their normal workload. However, the structure and resources that come with the Learning Community validates state team members’ work on immediate postpartum LARC. As one state team member explained, “I think just the validity of having [the learning community] as a shell around the effort…That we’re working with an ASTHO Learning Community…as a framework is going to lend so much credibility to this work, rather than [being] just a loosely affiliated team. We’ve already jumped way ahead of where I thought we would be at this point in time.”
3. *Preparing for potential challenges and opportunities*: Implementation of a statewide immediate postpartum LARC policy is not linear. The Learning Community includes states that initiated their reimbursement policies at various time points, and the states are at varying stages of implementation. Oftentimes, this means that one state is progressing faster than others within one Learning Community domain, but slower than others in another domain. Learning from each other’s successes and failures helps the states prepare for potential future challenges and opportunities. This is exemplified by the experience of one state team member, who said, “I think the learning collaborative…started us thinking about things that maybe we hadn’t thought about that you start to realize more and more as you get deeper into making progress. I think that brought to light things that we should be thinking about and ways to think about them and the realization that there are lots of different approaches as well. I think that’s been really valuable.”

### Implementation strategies mobilized by the Learning Community

Above and beyond the Learning Community serving as an implementation strategy on its own, the qualitative data demonstrated that the structure of this learning collaborative helps support and integrate six other implementation strategies described by the ERIC project [14]. Throughout the rest of this section, we will provide examples from our interviews with the state teams to demonstrate how this is the case for each strategy. The definitions for the implementation strategies described here are found in Table 2.

**Table 2:** Definitions and examples of the implementation strategies described

Implementation Strategy	Definition^a^	Example from Learning Community
Create a learning collaborative	Facilitate the formation of groups of providers or provider organizations and foster a collaborative learning environment to improve implementation of the clinical innovation	Creation of the ASTHO Immediate Postpartum LARC Learning Community
Organize clinician implementation team meetings	Develop and support teams of clinicians who are implementing the innovation and give them protected time to reflect on the implementation effort, share lessons learned, and support one another’s learning	In-person meetings provide a unique opportunity for the state teams to work on their immediate postpartum LARC efforts, including creating action plans to prioritize their activities.
Conduct educational meetings	Hold meetings targeted toward different stakeholder groups to teach them about the clinical innovation	Learning Community virtual learning sessions, which feature guest experts, provide a forum for ongoing education.
Facilitation	A process of interactive problem solving and support that occurs in a context of a recognized need for improvement and a supportive interpersonal relationship	ASTHO facilitates virtual learning sessions and in-person meetings, which incorporate problem solving and foster the sense of a supportive team across the country.
Promote network weaving	Identify and build on existing high-quality working relationships and networks within and outside the organization to promote information sharing, collaborative problem-solving, and a shared vision/goal related to implementing the innovation	- State teams include representatives from Medicaid and the state health department, and many of these agencies do not traditionally work together. - State teams have many opportunities, including through the in-person meetings and virtual learning sessions, to network with other state teams. - In-person meetings and virtual learning sessions include national partner agencies, which states can collaborate with and learn from.
Provide ongoing consultation	Provide ongoing consultation with one or more experts in the strategies used to support implementing the innovation	Experts participate in the in-person meetings and virtual learning sessions, and state teams are able to follow up for more in-depth assistance.
Distribute educational materials	Distribute educational materials in person, by mail, and/or electronically	States developed provider bulletins, patient guides, etc., and then shared them with ASTHO, who then shares the materials with the Learning Community.

^a^The implementation strategies and definitions in this table were published by the Expert Recommendations for Implementing Change (ERIC) project [14]

#### Organize clinician implementation team meetings

Implementation efforts for clinical activities can be strengthened by providing time to clinicians involved to reflect on their efforts, share lessons learned, and support each other [14]. While most of the state team members are not clinicians, the in-person Learning Community meetings provide a unique opportunity for the state implementation teams, including the physician champions, to work on their immediate postpartum LARC efforts. These meetings happen once per year for 2 days and are highly valued by state team members because of the protected time they have, including time set aside for creating action plans to prioritize their activities. This point is illustrated by one state team member, who explained, “At our first [in-person Learning Community] meeting, our physician champion was there…She’s probably about two and a half hours away from the rest of our team. [The rest of our team] interacts almost on a daily basis. We’re in the same space on the same floor of the same building. [Our physician champion]…as you can imagine, is really busy. It gave us time to really think through some things and talk. I think it was great to have her get to know us personally a little bit better, the whole team, and what our mission and vision was…At work we wear a lot of different hats. When we’re at the [in-person Learning Community] meetings, you can’t be distracted by other things. You can actually focus on that.”

#### Conduct educational meetings

The Learning Community hosts quarterly virtual learning sessions, which provide a forum for ongoing education. These virtual learning sessions feature guest experts on topics related to the eight Learning Community domains listed in Table 1. The technology used for these sessions includes a chat box for participants to communicate with each other during the live presentation, poll questions for participants to give instant feedback to presenters and answer topic-specific questions posed by ASTHO and partner agencies, and recording of the presentation, allowing for the learning sessions to be archived and made publicly available [22]. The state teams reported that the virtual learning sessions have been helpful in supporting their immediate postpartum LARC work. One example of the utility of the virtual learning sessions is explained by a state team member, “One of the things I find helpful is the fact that [the virtual learning sessions] are archived. I find in some of the presentations they’re a little further along than my thinking is. It’s nice to be able to go back when I get to that point to refer back and review that information.”

#### Facilitation

ASTHO facilitates the virtual learning sessions and in-person meetings, incorporating interactive problem solving while fostering the sense of a supportive team across the country. This sense of problem solving and support among the Learning Community teams is exemplified by this state team member, who said, “I think the connections to folks outside of the actual [virtual learning sessions] or [in-person] meetings has been very helpful so that we can get additional information if we needed it… I think that right now we’re even dealing with some issues with reimbursement that are very reminiscent of the things that we heard [another state] say [during a Learning Community activity]. Even having had that relationship and having seen that presentation [about the other state’s issues with reimbursement], we find ourselves having a similar problem here right now. It’s nice to know that you’re not alone…At the same time it is nice to be able to commiserate with others. I think that there’s value in that and hopefully it will help speed up the corrective actions that we can take because we can learn from others.”

#### Promote network weaving

The Learning Community provides opportunities for state teams to network within their own state in novel ways, network with other state teams, and network with national partner organizations. In addition to requiring an internal partnership between each state’s Medicaid agency and health department for Learning Community participation, ASTHO also helps connect teams across states and with other partners when technical assistance needs arise.

Learning Community state teams include representatives from the state Medicaid agency and the state health department, providing an opportunity for state agencies to begin working together if they have not traditionally done so. This partnership is strongly encouraged by ASTHO and results in state teams often growing their internal networks. One example of this is explained by a state team member who works for a state health department. This state had trouble approving an immediate postpartum LARC Medicaid policy, in part because the effort was solely based within the state health department. When the state joined the Learning Community, a representative from the state’s Medicaid agency joined the team working on this effort. The state team member explained, “For me, the most beneficial piece of [the Learning Community] has been meeting [the Medicaid representative]. [She] was not initially a part of our team. It took a while for us to get to [her] and to have her join our team.”

Learning Community participants have many opportunities to network across state teams, including through in-person meetings and virtual learning sessions. All the state teams highlighted the importance of this during the interviews, especially learning what other teams are doing through the official Learning Community activities, then following up informally with each other to share more details. As one state team member explained, “[My state] and [another state] have had lots of offline conversations about the various LARC programs and LARC training programs. That’s an important piece [of the Learning Community], some of those connections made and offline conversations.”

The in-person Learning Community meetings and the virtual learning sessions often include representatives from national partner agencies and organizations. This allows state team members to network with people working on similar issues at the national level. The value of this is explained by one state team member, who said, “I think having not only the states represented, but some of the other organizations, from the [American Congress of Obstetricians and Gynecologists] and [Centers for Medicare and Medicaid Services] and [Association of Maternal and Child Health Programs] and all the other organizations that were [at the in-person Learning Community meeting]. I think that was really valuable so that [the national organizations] can hear some of what’s going on in states and states can pick their brains a little bit, too. It’s also helpful to kind of get on the same page so that different organizations aren’t going down the different paths.”

#### Provide ongoing consultation

State team members and individuals from national agencies who are experts in areas related to one (or more) of the eight Learning Community domains participate in the in-person meetings and virtual learning sessions to share their expertise with the Learning Community participants. State teams are able to follow up with these experts for more in-depth assistance as the need arises. As one state team member explained, “I took all the stuff from this toolkit [I am developing] from [the toolkit another state Medicaid representative developed]. We’re sending the executive director of our collaborative out to [the other state] to get some mentorship from [the Medicaid representative].”

#### Distribute educational materials

The structure of the Learning Community helps with the distribution of educational materials related to immediate postpartum LARC. Several states have developed provider bulletins and patient informational handouts related to immediate postpartum LARC. These states have the option of sharing their materials with ASTHO, which in turn shares the materials with the other states in the Learning Community. Some of these materials are also made available on the ASTHO website for public viewing [23]. The state teams reported that this is helpful because they can build on the work of others and tailor materials to the needs of their states, instead of each starting from the beginning. One state team member described the way she uses the materials available, “I have information [to share with hospitals] in an organized folder that has a very brief PowerPoint on why immediate postpartum LARCs are encouraged, information on billing Medicaid, information on a sample hospital policy, sample consents, information for pharmacy, the videos for the physicians…A lot of stuff that I got from the ASTHO learning collaborative are the pieces that I use. That is a [benefit of the Learning Community]…references and resources.”

## Discussion

Qualitative analysis of semi-structured interviews with the 13 states in the Learning Community demonstrated that this learning collaborative is an important strategy for states implementing this Medicaid policy change around the USA. Further, this learning collaborative serves as a meta-strategy for implementation. In other words, the activities of the Learning Community, including the yearly, 2-day in-person meeting and the quarterly virtual learning sessions, help integrate six other implementation strategies described by the ERIC project [14]: organize clinician implementation team meetings, conduct educational meetings, facilitation, promote network weaving, provide ongoing consultation, and distribute educational materials.

For a clinical innovation as complex as immediate postpartum LARC, establishing a Medicaid reimbursement policy alone is insufficient to bring about widespread access to LARC devices during the delivery hospitalization [12]. As previous literature has suggested, successes in dissemination, implementation, and sustainment of evidence-based practices occur through the development and application of deliberate strategies that may be effective across many different clinical innovations and guidelines [24]. Results from this study suggest that implementation strategies, such as creating a learning collaborative, can help states facilitate implementation of policies designed to improve access to the most effective contraceptive methods in the immediate postpartum period as well as address barriers. In previous studies, implementation strategies have been organized into distinct clusters [25] and grouped according to where they fall in the implementation process [26]. Research teams have sought to test the effectiveness of implementation strategies alone or in combination [27]. To our knowledge, we are the first researchers to describe how one strategy might serve as a meta-strategy to bundle together several other strategies. It should be noted that this is different than what the researchers in the ERIC project proposed, as their implementation strategies are “discrete” and “conceptually distinct” [14, 25]. However, this analysis revealed that the Learning Community, as implemented by ASTHO and its partner agencies, demonstrates how creating a learning collaborative could be a unifying strategy to support the implementation of evidence-based public health practices.

The key steps necessary for implementing immediate postpartum LARC policies take place at many levels within the US context. At the national level, the Learning Community brings together teams from several states and federal partners to share strategies and lessons learned to address implementation challenges. At the state level, each team has worked to establish the Medicaid reimbursement policy in its state and then supports implementation of this clinical innovation (i.e., the LARC insertion) by employing statewide strategies within the eight domains listed in Table 1. While the state teams play a large role in supporting implementation, immediate postpartum LARC insertion ultimately takes place in birthing facilities. Future studies should evaluate implementation strategies that can be employed at the facility level, especially related to the Learning Community domains of outreach, provider training, and stocking and supply.

This study has several important strengths and limitations. First, most state team members were interviewed together, which was both a strength and limitation. We had the opportunity to observe the teams’ various planning and interaction styles, and their responses to our questions were enhanced because they built upon each other. However, we may not have captured the more challenging aspects of their work as a team because individuals may have felt uncomfortable sharing interpersonal challenges affecting the team’s implementation work. Similarly, interviews with state teams were conducted over the telephone. This enabled us to conduct interviews with all 13 state teams, but it prevented us from observing non-verbal communication cues that may have been important for this study. Additionally, the purpose of our interviews was not to identify which of the implementation strategies from the taxonomy proposed in the ERIC project were being applied. Therefore, while we found that the Learning Community served as a meta-strategy to help integrate six other implementation strategies, this list may not be exhaustive. Further, we did not measure state teams’ progress over time or implementation outcomes. Finally, the Learning Community was not designed as part of an experiment to study the effectiveness of implementation strategies, and we do not have comparable data from state teams that are not participating in the Learning Community. However, this study adds to the implementation science literature by describing the benefits perceived by the state teams participating in the Learning Community as they relate to a commonly used taxonomy of implementation strategies [14]. Conceptualizing the Learning Community as a meta-strategy may inform other implementation efforts that are considering forming a learning collaborative to promote the adoption or sustainment of evidence-based practices. Future implementation research should use Proctor and colleagues’ framework to comprehensively specify each of the implementation strategies packaged within learning collaboratives [13], then empirically test the effect of individual implementation strategies, and the effect of the interaction between multiple implementation strategies within a learning collaborative, on implementation outcomes.

The work performed by state teams in the Learning Community has important public health implications. First, the relationships developed through this collaborative have the capacity to extend into other public health initiatives, creating an added benefit for state teams. Additionally, Medicaid covers approximately 50% of all deliveries in the USA [28], and Medicaid policies are often adopted later by private insurance plans. Therefore, as states participating in the Learning Community learn to successfully implement the immediate postpartum LARC reimbursement policy, including overcoming the additional barriers to full implementation, there is the potential to increase access to immediate postpartum LARC for all patients who desire it.

## Conclusions

Convening a multi-state learning collaborative is a promising approach for facilitating the implementation of new reimbursement policies for evidence-based practices complicated by systems challenges. By providing a structure for integrating several complementary implementation strategies, the Learning Community serves as a “meta-strategy” for supporting implementation.

## Abbreviations

ASTHO: Association of State and Territorial Health Officials
CDC: Centers for Disease Control and Prevention
DRG: Diagnosis-Related Group
ERIC: Expert Recommendations for Implementing Change
IUD: Intrauterine device
LARC: Long-acting reversible contraception
